# How transformational leadership is associated with primary and secondary school teachers’ social–emotional competence: a multiple mediation analysis

**DOI:** 10.3389/fpsyg.2026.1777257

**Published:** 2026-06-03

**Authors:** Qilan Li, Jinwei Tu, Biao Zeng

**Affiliations:** 1 College of Education, China Conservatory of Music, Beijing, China; 2 School of Music, Anhui Normal University, Wuhu, Anhui, China; 3 Normal College, Jimei University, Xiamen, Fujian, China

**Keywords:** Chinese teachers, multiple mediation, primary and secondary school teachers, social–emotional competence, transformational leadership

## Abstract

This study examined indirect association patterns between Transformational Leadership and primary and secondary school teachers’ Social–Emotional Competence, with supplementary analyses exploring whether these patterns varied across leadership dimensions. Drawing on Ecological Systems Theory and the Prosocial Classroom Model, data were collected from 760 primary and secondary school teachers and analyzed using a multiple mediation model. Transformational Leadership was significantly and positively associated with teachers’ Social–Emotional Competence. In the overall model, the indirect association via Teaching Efficacy was the strongest; the pathway via Teacher-Student Relationship was significant but small, whereas the pathway via School Climate was not significant. Supplementary dimension-specific analyses showed that the relative prominence of these pathways varied across leadership dimensions. Overall, the findings indicate that Teaching Efficacy was the most prominent correlate in the estimated indirect association between Transformational Leadership and teachers’ Social–Emotional Competence, while School Climate showed a less consistent pattern. These results highlight the relevance of both school contextual resources and teachers’ confidence-related psychological resources for understanding teachers’ social–emotional competence.

## 1 Introduction

At the end of the 20th century, the field of education experienced an “affective turn” (Huang, 2024). As implementers of affective education, primary and secondary school teachers’ Social–Emotional Competence (SEC) is widely regarded as important for building a high-quality teaching workforce and for helping individuals adapt to society. In general, teacher SEC refers to teachers’ capacity, developed through interactions with their environment, to manage their own emotions effectively, establish positive interpersonal relationships, and make responsible decisions (CASEL, 2013). Teacher SEC has been associated with multiple aspects of the educational ecosystem. For teachers themselves, a higher level of SEC has been associated with lower burnout (Tian et al., 2021a), higher occupational well-being (Tian et al., 2021b), stronger Teaching Efficacy, and more favorable professional development outcomes (Du and Liu, 2024). For students, teachers’ SEC has also been associated with students’ social–emotional development and may be related to more harmonious Teacher-Student Relationship and the development of students’ positive values (Jennings and Greenberg, 2009; Zhang et al., 2022). As “significant others” within school organizations and as change agents, school leaders may represent one of the organizational factors most closely associated with teachers’ occupational psychological development. Leaders with a Transformational Leadership style are often described as attending to employees’ emotional changes, supporting the fulfillment of their needs, and maintaining positive and proactive affective connections with employees. Accordingly, Transformational Leadership has been associated with higher levels of commitment and trust, as well as with a more supportive School Climate for employees’ work and development (Sui et al., 2012), which may in turn be associated with teachers’ SEC. However, several gaps remain at the empirical level. First, direct evidence on how Transformational Leadership is associated with teachers’ Social–Emotional Competence remains limited. Second, prior studies have more often examined potentially relevant correlates in isolation or in separate models, rather than testing whether individual-level, relational, and school-contextual correlates differ in prominence when considered simultaneously. Third, Transformational Leadership has usually been treated as an overall construct, leaving potential heterogeneity across its subdimensions insufficiently examined. As a result, it remains unclear not only whether Transformational Leadership is associated with teachers’ Social–Emotional Competence, but also which types of correlates appear more or less prominent within a unified model.

Against this backdrop, the present study employed a questionnaire-based design to examine the association between Transformational Leadership and primary and secondary school teachers’ Social–Emotional Competence. Guided by Ecological Systems Theory (Bronfenbrenner, 1979) and the Prosocial Classroom Model, the study focused on three potentially relevant correlates located at different levels of school life: Teacher-Student Relationship, Teaching Efficacy, and School Climate. Considering these variables within one model allowed a more direct comparison of their relative prominence while reducing the risk of interpreting each pathway in isolation. Supplementary dimension-specific analyses further explored whether the indirect association patterns varied across the four dimensions of Transformational Leadership.

## 2 Literature review

Building on these two complementary frameworks, the literature review first considers the association between Transformational Leadership and teachers’ Social–Emotional Competence, and then discusses Teacher-Student Relationship, Teaching Efficacy, and School Climate as classroom-relational, proximal individual, and broader school-contextual correlates in the proposed model.

### 2.1 The association between transformational leadership and primary and secondary school teachers’ social–emotional competence

Transformational Leadership is generally characterized by its emphasis on employees’ innovation, personal development, and social relationships. In the present study, its association with teachers’ Social–Emotional Competence is interpreted within the broader ecological and relational conditions of school life. As a leadership style, Transformational Leadership has been associated with more positive interactions with organizational members, as well as with teachers’ confidence, inspiration, creativity, and intrinsic motivation, all of which may be relevant to teachers’ psychological functioning and work experiences (Tian et al., 2021a; Tian et al., 2021b). Transformational Leadership comprises four dimensions—Moral Modeling, Articulate Vision, Charisma, and Individualized Consideration (Li and Shi, 2005; Wang and Howell, 2010)—and may represent an important external contextual factor associated with primary and secondary school teachers’ Social–Emotional Competence.

From the perspective of the Prosocial Classroom Model, teachers’ social–emotional functioning is embedded in the relational and contextual conditions of schools and classrooms (Jennings and Greenberg, 2009). In this sense, Transformational Leadership is relevant here because it is associated with the relational climate and developmental support surrounding teachers’ everyday work. Within more supportive school contexts, teachers may report greater confidence, optimism, and intrinsic motivation, and these tendencies may also be associated with higher levels of Social–Emotional Competence (Xiong et al., 2023). Prior studies have also reported a positive association between Transformational Leadership and teachers’ Social–Emotional Competence (Tian et al., 2021b).

*Hypothesis 1 (H1)*: Transformational Leadership is positively associated with teachers’ Social-Emotional Competence.

### 2.2 The indirect role of teacher-student relationship

Teacher-Student Relationship is an egalitarian dialogical relationship between “I” and “you,” in which teachers and students—each as independent spiritual subjects—enter one another’s horizons on the basis of mutual respect and trust, achieving mutual understanding and equal communication (Shao, 2005). In the present study, Teacher-Student Relationship is treated as a classroom-relational correlate of teachers’ Social–Emotional Competence. From the perspective of the Prosocial Classroom Model, teachers’ social–emotional functioning is embedded in ongoing classroom interactions, and Teacher-Student Relationship represents a proximal relational context closely associated with teachers’ Social–Emotional Competence. Negative exchanges between teachers and students may be associated with teachers’ affective experiences, whereas more positive Teacher-Student Relationships may be associated with greater emotional support, better interaction quality, and stronger emotional efficacy (Hu et al., 2025; Pedditzi et al., 2021; Yan et al., 2024). Prior empirical evidence has also reported a positive association between Teacher-Student Relationship and teachers’ Social–Emotional Competence (Zhang et al., 2022).

Transformational Leadership may also be indirectly associated with teachers’ Social–Emotional Competence through Teacher-Student Relationship. From an ecological perspective, leadership-related conditions at the school level may be associated, albeit indirectly, with teachers’ relational experiences in classrooms. When teachers perceive the school atmosphere as characterized by trust, support, and respect, they may also report more caring and collaborative relationships with students. Such classroom relational experiences may also be associated with teachers’ emotional feedback, interpersonal experiences, and Social–Emotional Competence.

*Hypothesis 2 (H2)*: Teacher-Student Relationship is expected to mediate the positive association between Transformational Leadership and teachers’ Social-Emotional Competence.

### 2.3 The indirect role of teaching efficacy

Teaching Efficacy refers to teachers’ judgments of their capability to effectively bring about desired instructional outcomes, such as engaging students, managing classroom demands, and supporting student learning (Tschannen-Moran and Hoy, 2001). Although Teaching Efficacy and teachers’ Social–Emotional Competence are closely related, they are theoretically distinct constructs. Teaching Efficacy is rooted in self-efficacy theory and primarily reflects a context-specific belief about what teachers think they can accomplish in teaching situations (Bandura, 1997; Tschannen-Moran and Hoy, 2001). By contrast, Social–Emotional Competence refers to a broader set of emotional and interpersonal capacities, including emotion regulation, social awareness, relationship skills, and responsible decision-making in educational contexts (Jennings and Greenberg, 2009; Schonert-Reichl, 2017). Emotion regulation in work-related social interactions has also been theorized as an important factor associated with work strain and interpersonal functioning (Côté, 2005). Accordingly, Teaching Efficacy reflects a more proximal self-appraisal of instructional capability, whereas Social–Emotional Competence reflects broader psychosocial functioning in managing the emotional and relational demands of teaching. A teacher may feel efficacious in delivering instruction without necessarily possessing equally strong emotional regulation or relationship-management skills; conversely, a teacher may demonstrate sound social–emotional skills while still doubting their instructional effectiveness. From this perspective, Teaching Efficacy may be understood as a proximal individual-level appraisal that is associated with, but not reducible to, teachers’ broader Social–Emotional Competence.

First, Teaching Efficacy may represent an important proximal individual-level correlate of teachers’ Social–Emotional Competence. As a context-specific self-appraisal of instructional capability, Teaching Efficacy reflects how teachers evaluate their capacity to manage classroom demands. Teachers with stronger Teaching Efficacy may report greater confidence and persistence when responding to instructional challenges, and these tendencies may also be associated with more adaptive emotion regulation and interpersonal functioning in educational settings. Consistent with this reasoning, prior research has reported associations between teachers’ efficacy beliefs and their social–emotional functioning and related emotional outcomes (Cheng et al., 2022; Zhang et al., 2022).

Second, Transformational Leadership may be indirectly associated with teachers’ Social–Emotional Competence through Teaching Efficacy. In the present study, Teaching Efficacy is conceptualized as a proximal individual-level correlate of teachers’ Social–Emotional Competence. When teachers report more supportive leadership contexts, they may also report stronger efficacy-related beliefs, and these beliefs are examined as one possible correlate of teachers’ Social–Emotional Competence in the present model. From this perspective, Teaching Efficacy is examined as one proximal correlate within the broader association between Transformational Leadership and teachers’ Social–Emotional Competence.

*Hypothesis 3 (H3)*: Teaching Efficacy is expected to mediate the positive association between Transformational Leadership and teachers’ Social-Emotional Competence.

### 2.4 The indirect role of school climate

A healthy School Climate is widely regarded as important for the overall development of a school (Halpin, 1966). School Climate refers to relatively enduring characteristics of the school environment that can be experienced by its members and that may be associated with their psychological states and behaviors (Pu et al., 2023). In the present study, School Climate is treated as a broader school-contextual correlate of teachers’ Social–Emotional Competence. From both ecological and prosocial classroom perspectives, teachers’ social–emotional functioning may be associated with the wider relational and contextual environment in which teaching occurs. A positive and inclusive School Climate may be associated with emotional support, psychological safety, and more stable affective experiences among teachers (Pu et al., 2023). Empirical evidence has also shown that School Climate is associated with teachers’ Social–Emotional Competence (Zhang et al., 2022).

Transformational Leadership may also be indirectly associated with teachers’ Social–Emotional Competence through School Climate. By articulating a shared vision and shaping interactions among school members, Transformational Leadership may be associated with a more supportive organizational atmosphere characterized by trust, collaboration, and care (Leithwood, 1992; Zhang and Mao, 2022). Such broader school conditions may in turn be associated with teachers’ emotional stability and social functioning in school life.

*Hypothesis 4 (H4)*: School Climate is expected to mediate the positive association between Transformational Leadership and teachers’ Social-Emotional Competence.

In summary, although prior research has reported that Transformational Leadership, School Climate, Teacher-Student Relationship, and Teaching Efficacy are important variables associated with teachers’ Social–Emotional Competence, most studies have tended to treat Transformational Leadership as an overall construct, which may obscure potentially different patterns associated with its subdimensions. According to the localized Transformational Leadership framework proposed by Li and Shi (2005), Moral Modeling emphasizes the leader’s role as a moral exemplar, Articulate Vision highlights spiritual inspiration and value-based appeal, Charisma focuses on personal attractiveness, and Individualized Consideration underscores micro-level emotional support. The four dimensions of Transformational Leadership emphasize different aspects of leadership behavior; accordingly, it is reasonable to expect that their indirect association patterns with Teacher-Student Relationship, Teaching Efficacy, and School Climate may not be fully homogeneous. Such variation is theoretically plausible because different leadership dimensions may align to different extents with the relational and contextual conditions captured in the model. At the same time, any such dimensional variation should be interpreted cautiously, as the overall model remains the primary analytic focus of the present study.

*Hypothesis 5 (H5)*: The four dimensions of Transformational Leadership (Moral Modeling, Articulate Vision, Charisma, and Individualized Consideration) may show different indirect association patterns with teachers’ Social-Emotional Competence through School Climate, Teacher-Student Relationship, and Teaching Efficacy.

**Figure 1:**
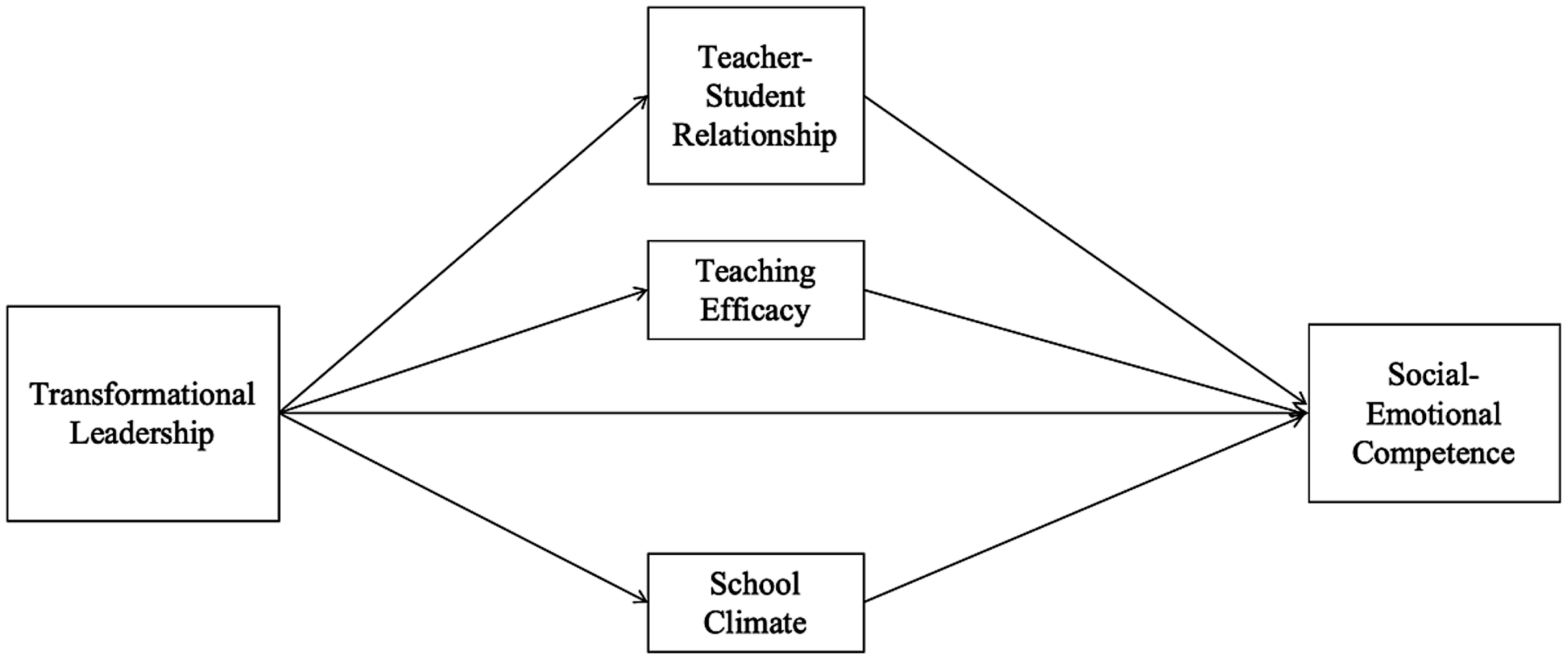
Hypothesized research model linking transformational leadership to teachers’ social–emotional competence through teacher-student relationship, teaching efficacy, and school climate.

Based on the theoretical reasoning above, we specified a multiple mediation theoretical model (Figure 1) to examine the indirect association patterns linking Transformational Leadership with teachers’ Social–Emotional Competence at both the overall level and the subdimension level. At the same time, prior findings on contextual factors related to teachers’ Social–Emotional Competence have not always been fully consistent, particularly for broader school-level conditions such as School Climate, whose associations may vary depending on sample characteristics, analytical level, and the inclusion of more proximal individual-level variables.

## 3 Methods

### 3.1 Participants and procedure

This study used a convenience sampling strategy to recruit primary and secondary school teachers from multiple cities, including Beijing, Shijiazhuang, Langfang, Tangshan, and Jinzhou. These cities were selected based on accessibility and existing institutional contacts that facilitated school recruitment rather than through a random sampling procedure. With approval from school administrators and informed consent from participating teachers, questionnaires were distributed through both online and offline formats. The use of both formats was intended to increase accessibility and participation across schools with different organizational conditions. The two formats contained identical questionnaire content and response structure, and the same data-screening criteria were applied to both. The survey emphasized voluntary participation, confidentiality, and anonymous completion. A total of 826 questionnaires were distributed. The inclusion criteria were as follows: participants had to be currently employed primary or secondary school teachers, voluntarily agree to participate, and complete the core study measures. After excluding invalid responses due to patterned responding and missing data, 760 valid questionnaires were retained for the formal analyses, yielding an effective response rate of 92.01%. The final sample included 211 male teachers (27.8%) and 549 female teachers (72.2%). The mean age of participants was 36.65 years (SD = 10.74). No formal *a priori* sample size calculation was conducted. However, the final sample size of 760 was deemed adequate for the planned regression and bootstrap mediation analyses. The study protocol was approved by the Ethics Committee of Anhui Normal University (Approval No. AHNU-ET2024166), and all participants provided written informed consent.

### 3.2 Measures

#### 3.2.1 Transformational leadership

Transformational Leadership was assessed using the Transformational Leadership Questionnaire developed by Li and Shi (2005). This instrument was developed in accordance with Chinese cultural characteristics and is suitable for the Chinese educational context. It comprises four dimensions—Moral Modeling, Articulate Vision, Charisma, and Individualized Consideration—with a total of 26 items. Responses were rated on a 5-point Likert scale ranging from “strongly disagree” to “strongly agree,” based on teachers’ actual experiences. Higher scores indicate more positive leadership behaviors exhibited by school leaders. Reliability analysis showed that the overall Cronbach’s *α* for the Transformational Leadership scale was 0.965.

#### 3.2.2 Teacher-student relationship

Teacher-Student Relationship was measured using the Teacher-Student Relationship Scale developed by Wang et al. (2001). The scale was developed to reflect Chinese cultural characteristics and is suitable for the Chinese educational context. It comprises three dimensions—conflict, closeness, and responsiveness—with a total of 22 items. Responses were rated on a 5-point Likert scale ranging from “strongly disagree” to “strongly agree,” based on teachers’ actual experiences. Higher scores indicate better Teacher-Student Relationship. Reliability analysis showed that the overall Cronbach’s *α* for the Teacher-Student Relationship scale was 0.841.

#### 3.2.3 Teaching efficacy

Teaching Efficacy was assessed using the Teacher Efficacy Scale developed by Tschannen-Moran and Hoy (2001). This scale has been used in the Chinese educational context and has demonstrated good reliability and validity (Zhang et al., 2022). The instrument includes three dimensions—Efficacy for instructional strategies, Efficacy for classroom management, and Efficacy for student engagement—with a total of 24 items. Responses were rated on a 5-point Likert scale ranging from “strongly disagree” to “strongly agree,” based on teachers’ actual experiences. Higher scores indicate higher levels of Teaching Efficacy. Reliability analysis indicated that the overall Cronbach’s *α* for the Teaching Efficacy scale was 0.944.

#### 3.2.4 School climate

School Climate was measured using the Organizational Climate Description Questionnaire (OCDQ) developed by Hoy and Clover (1986). The scale comprises six dimensions: Supportive Behavior, Directive Behavior, Restrictive Behavior, Collegial Behavior, Intimate Behavior, and Disengaged Behavior, with a total of 42 items. Responses were rated on a 5-point Likert scale ranging from “strongly disagree” to “strongly agree,” based on teachers’ actual experiences. Higher scores indicate a more positive School Climate as perceived by teachers. Reliability analysis showed that the overall Cronbach’s α for the School Climate scale was 0.844.

#### 3.2.5 Social–emotional competence

Teachers’ Social–Emotional Competence was assessed using a revised version of the Primary and Secondary School Teachers’ Social–Emotional Competence Scale developed by the Beijing Normal University research team (Zhang et al., 2022). The scale was developed to reflect Chinese cultural characteristics and is suitable for the Chinese educational context. It includes six dimensions—self-awareness, self-management, other-awareness, other-management, group-awareness, and group-management—with a total of 23 items. In this study, the original item in the group-awareness dimension (“I identify with the school’s culture and rules and regulations”) was revised into two items—“I identify with the school’s culture” and “I endorse the school’s rules and regulations”—to more precisely capture the intended meaning of the item. Responses were rated on a 5-point Likert scale ranging from “strongly disagree” to “strongly agree,” based on teachers’ actual experiences. Higher scores indicate higher levels of Social–Emotional Competence. Reliability analysis indicated that the overall Cronbach’s *α* for the Social–Emotional Competence scale was 0.934.

### 3.3 Statistical analysis

All statistical analyses were conducted using SPSS 26.0 and Mplus. The analytical procedure comprised four components. First, common method bias and reliability were evaluated. Harman’s single-factor test was used as a preliminary diagnostic check for potential common method bias, and internal consistency reliability for each scale was assessed using Cronbach’s α. Second, descriptive statistics and correlation analyses were conducted to summarize the central tendencies and bivariate associations among the study variables. Third, because Teaching Efficacy and Social–Emotional Competence were highly correlated, supplementary analyses were conducted to examine their empirical distinctiveness and the robustness of the focal indirect association via Teaching Efficacy. These analyses included item-level CFA model comparisons, an additional model-comparison test, and a focused latent-variable mediation robustness check. The main text summarizes only the core conclusions, whereas the detailed model comparisons, fit indices, and robustness-check results are reported in the Supplementary material. Fourth, multiple linear regression and mediation analyses were conducted to estimate the direct and indirect associations among Transformational Leadership, Teacher-Student Relationship, Teaching Efficacy, School Climate, and Social–Emotional Competence. Indirect effects were examined using Hayes’ SPSS PROCESS macro (Model 4) with a bootstrap procedure (5,000 resamples; 95% confidence intervals).

## 4 Results

### 4.1 Common method bias test

Harman’s single-factor test was used only as a preliminary diagnostic check for potential common method bias. Specifically, all items from the Transformational Leadership, School Climate, Teacher-Student Relationship, Teaching Efficacy, and Social–Emotional Competence scales were entered into an exploratory factor analysis, and the proportion of variance explained by the first extracted factor was compared with the conventional 40% threshold (Podsakoff et al., 2003). The results indicated that 28 factors with eigenvalues greater than 1 were extracted, and the first factor accounted for 27.261% of the total variance (Table 1), which is below the conventional criterion. However, this result should be interpreted only as a preliminary diagnostic indication rather than as evidence that common method bias was absent. Because all variables were collected through same-source self-report measures at a single time point, Harman’s single-factor test provides only limited evidence regarding common method bias and cannot meaningfully rule out method-related inflation of the observed associations.

**Table 1:** Harman’s single-factor test.

Component	Total variance explained					
	Initial eigenvalues			Extraction sums of squared loadings		
	Total	% of variance	Cumulative %	Total	% of variance	Cumulative %
1	37.348	27.261	27.261	37.348	27.261	27.261
2	9.359	6.831	34.093	9.359	6.831	34.093
3	8.543	6.236	40.329	8.543	6.236	40.329
4	2.764	2.017	42.346	2.764	2.017	42.346
5	2.517	1.837	44.183	2.517	1.837	44.183
6	1.915	1.398	45.581	1.915	1.398	45.581
…	…	…	…	…	…	…
28	1.028	0.750	67.494	1.028	0.750	67.494
29	0.996	0.727	68.221			

### 4.2 Descriptive statistics

Table 2 presents the means (M), standard deviations (SD), Pearson correlation coefficients, skewness, and kurtosis for Transformational Leadership, School Climate, Teacher-Student Relationship, Teaching Efficacy, and Social–Emotional Competence. The skewness values ranged from −0.609 to −0.088, and the kurtosis values ranged from −0.402 to 2.561, indicating that the distributions of the main study variables were within acceptable ranges for the planned analyses. In addition, the Durbin—Watson statistic was 1.949, indicating no serious violation of the independence assumption, and residual plots did not indicate any serious heteroscedasticity. The results indicated that Transformational Leadership was significantly and positively correlated with Social–Emotional Competence (*r* = 0.619, *p* < 0.001), School Climate (*r* = 0.825, *p* < 0.001), Teacher-Student Relationship (*r* = 0.220, *p* < 0.001), and Teaching Efficacy (*r* = 0.580, *p* < 0.001). In addition, School Climate (*r* = 0.545, *p* < 0.001), Teacher-Student Relationship (*r* = 0.360, *p* < 0.001), and Teaching Efficacy (*r* = 0.862, *p* < 0.001) were also significantly and positively correlated with Social–Emotional Competence. All correlations were significant and in the expected direction, providing preliminary correlational support for the subsequent mediation analyses. Because the sample included both male and female teachers, group-specific descriptive statistics and independent-samples *t*-tests were conducted for the main study variables. As shown in Table 3, female teachers reported significantly higher scores than male teachers on Transformational Leadership, School Climate, Teacher-Student Relationship, Teaching Efficacy, and Social–Emotional Competence.

**Table 2:** Descriptive statistics and correlations among variables.

Variables	1	2	3	4	5
1. Transformational leadership	1				
2. School climate	0.825***	1			
3. Teacher-student relationship	0.220***	0.395***	1		
4. Teaching efficacy	0.580***	0.492***	0.352***	1	
5. Social–emotional competence	0.619***	0.545***	0.360***	0.862***	1
*M*	3.82	3.40	3.49	3.95	3.97
SD	0.62	0.34	0.46	0.50	0.51
Skewness	−0.593	−0.191	−0.088	−0.323	−0.609
Kurtosis	1.686	1.220	−0.402	2.544	2.561

*N* = 760. *p < 0.05, **p < 0.01, ***p < 0.001.

**Table 3:** Means, standard deviations, and independent-samples *t*-test results by gender.

Variables	Male (*n* = 211) M	Male SD	Female (*n* = 549) *M*	Female SD	*t*	*p*
1. Transformational leadership	3.688	0.699	3.871	0.577	−3.378	< 0.001
2. School climate	3.350	0.343	3.419	0.33	−2.559	0.011
3. Teacher-student relationship	3.350	0.463	3.549	0.451	−5.404	< 0.001
4. Teaching efficacy	3.868	0.557	3.976	0.466	−2.495	0.013
5. Social–emotional competence	3.876	0.622	4.011	0.457	−2.863	0.004

Male = 211; Female = 549. Independent-samples t tests were conducted by gender. Reported *t* and *p*-values are based on the appropriate equality-of-variance assumption according to Levene’s test.

Given the high correlation between Teaching Efficacy and Social–Emotional Competence (*r* = 0.862), supplementary analyses were conducted to examine whether the two constructs were empirically distinguishable. The CFA results showed that the two-factor models fit the data better than the one-factor model, although the overlap between the two constructs remained substantial. In addition, the model imposing a perfect association between Teaching Efficacy and Social–Emotional Competence fit the data significantly worse than the freely estimated two-factor model. Taken together, these findings suggest that Teaching Efficacy and Social–Emotional Competence were distinguishable but substantially overlapping. Detailed fit indices and model-comparison results are reported in Supplementary Tables S5, S6.

### 4.3 Multiple mediation analyses of the association between transformational leadership and social–emotional competence

We used Hayes (2013) SPSS PROCESS to examine the indirect effects of Teacher-Student Relationship, Teaching Efficacy, and School Climate in the association between Transformational Leadership and primary and secondary school teachers’ Social–Emotional Competence. Demographic variables—including gender, age, school type, professional title, school location type, and subject area—were entered as covariates. A bootstrap procedure with 5,000 resamples was applied to estimate the indirect effects, and 95% confidence intervals were used to evaluate statistical significance.

#### 4.3.1 Regression results for the multiple mediation model

**Figure 2:**
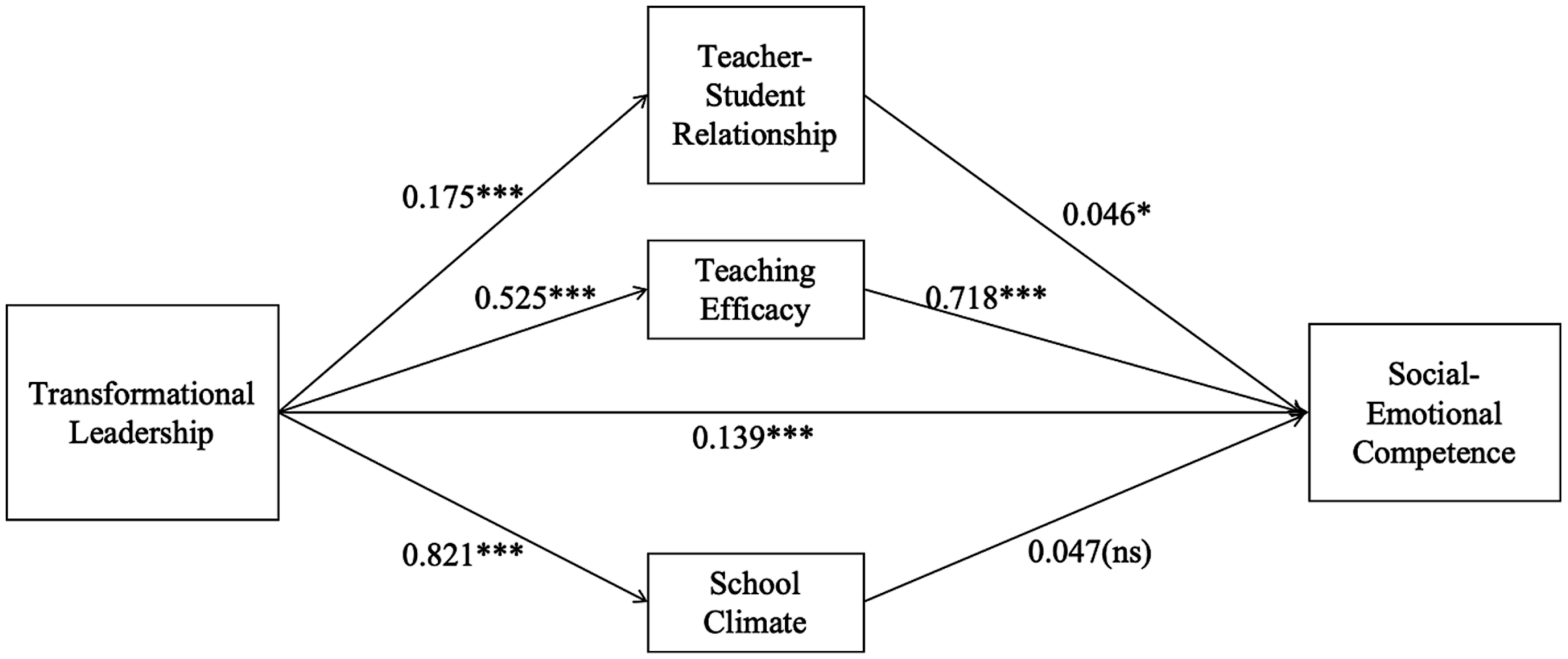
Multiple indirect associations between transformational leadership and social–emotional competence. *p < 0.05, **p < 0.01, ***p < 0.001.; ns = not significant.

We first examined the path coefficients. The standardized regression coefficients are presented in Figure 2 and Table 4. Transformational Leadership showed significant positive regression coefficients with Teacher-Student Relationship (*β* = 0.175, *p* < 0.001), Teaching Efficacy (*β* = 0.525, *p* < 0.001), and School Climate (*β* = 0.821, *p* < 0.001), indicating that Transformational Leadership was significantly associated with all three proposed mediators in the regression model.

**Table 4:** Standardized regression coefficients for the multiple mediation model.

Outcome variable	Predictor	*β*	SE	*t*	*p*
Teaching efficacy	Transformational leadership	0.525	0.024	17.637	<0.001
Teacher-student relationship	Transformational leadership	0.175	0.028	4.771	<0.001
School climate	Transformational leadership	0.821	0.012	37.892	<0.001
Social–emotional competence	Transformational leadership	0.139	0.029	3.98	<0.001
Social–emotional competence	Teaching efficacy	0.718	0.024	30.723	<0.001
Social–emotional competence	Teacher-student relationship	0.046	0.023	2.235	0.026
Social–emotional competence	School climate	0.047	0.052	1.37	0.171

Standardized regression coefficients are reported based on the PROCESS Model 4 output. Demographic covariates were included in all models but are not displayed for brevity.

When Transformational Leadership and the three mediators were simultaneously entered into the regression equation for Social–Emotional Competence, Teaching Efficacy (*β* = 0.718, *p* < 0.001) and Teacher-Student Relationship (*β* = 0.046, *p* < 0.05) showed significant positive regression coefficients, whereas the coefficient for School Climate did not reach statistical significance (*β* = 0.047, *p* > 0.05). In addition, the coefficient for the direct path from Transformational Leadership to Social–Emotional Competence remained statistically significant (*β* = 0.139, *p* < 0.001). These results indicate that, after accounting for the three proposed mediators, the direct path from Transformational Leadership to Social–Emotional Competence remained statistically significant in the model.

#### 4.3.2 Bootstrap results for the indirect effects

We further examined the statistical significance of the indirect effects using a bias-corrected, nonparametric percentile bootstrap procedure. The results are presented in Table 5. The total effect was 0.465, with a 95% confidence interval (CI) of [0.419, 0.512], indicating that the total effect was statistically significant. The direct effect was 0.115, with a 95% CI of [0.058, 0.172], indicating that the direct path remained statistically significant in the model. The total indirect effect was 0.350, with a 95% CI of [0.278, 0.421], indicating that the total indirect effect was also statistically significant.

**Table 5:** Decomposition of indirect effects between transformational leadership and social–emotional competence.

Effect pathway	Effect	Boot SE	95% CI Lower limit (LLCI)	95% CI Upper limit (ULCI)	Relative effect proportion (%)
Total effect	0.465	0.024	0.419	0.512	–
Direct effect	0.115	0.029	0.058	0.172	24.73%
Total indirect effect	0.350	0.036	0.278	0.421	75.27%
Mediating pathway 1: teacher-student relationship	0.007	0.004	0.0002	0.0150	1.44%
Mediating pathway 2: teaching efficacy	0.312	0.032	0.251	0.376	67.06%
Mediating pathway 3: school climate	0.032	0.025	−0.018	0.079	6.77%

Indirect effects are bootstrapped estimates (5,000 samples) with 95% bias-corrected confidence intervals. Regression coefficients in Figure 2 are standardized. For the Teacher-Student Relationship pathway, the unrounded lower confidence limit was slightly above zero (LLCI = 0.0002).

Three specific indirect effects were then examined. For Path 1 (via Teacher-Student Relationship), the estimated indirect effect was 0.007, with a 95% CI of [0.0002, 0.0150]. For Path 2 (via Teaching Efficacy), the estimated indirect effect was 0.312, with a 95% CI of [0.251, 0.376]. For Path 3 (via School Climate), the estimated indirect effect was 0.032, with a 95% CI of [−0.018, 0.079]. Based on the bootstrap confidence intervals, the indirect effects via Teacher-Student Relationship and Teaching Efficacy were statistically significant, whereas the indirect effect via School Climate was not.

**Figure 3:**
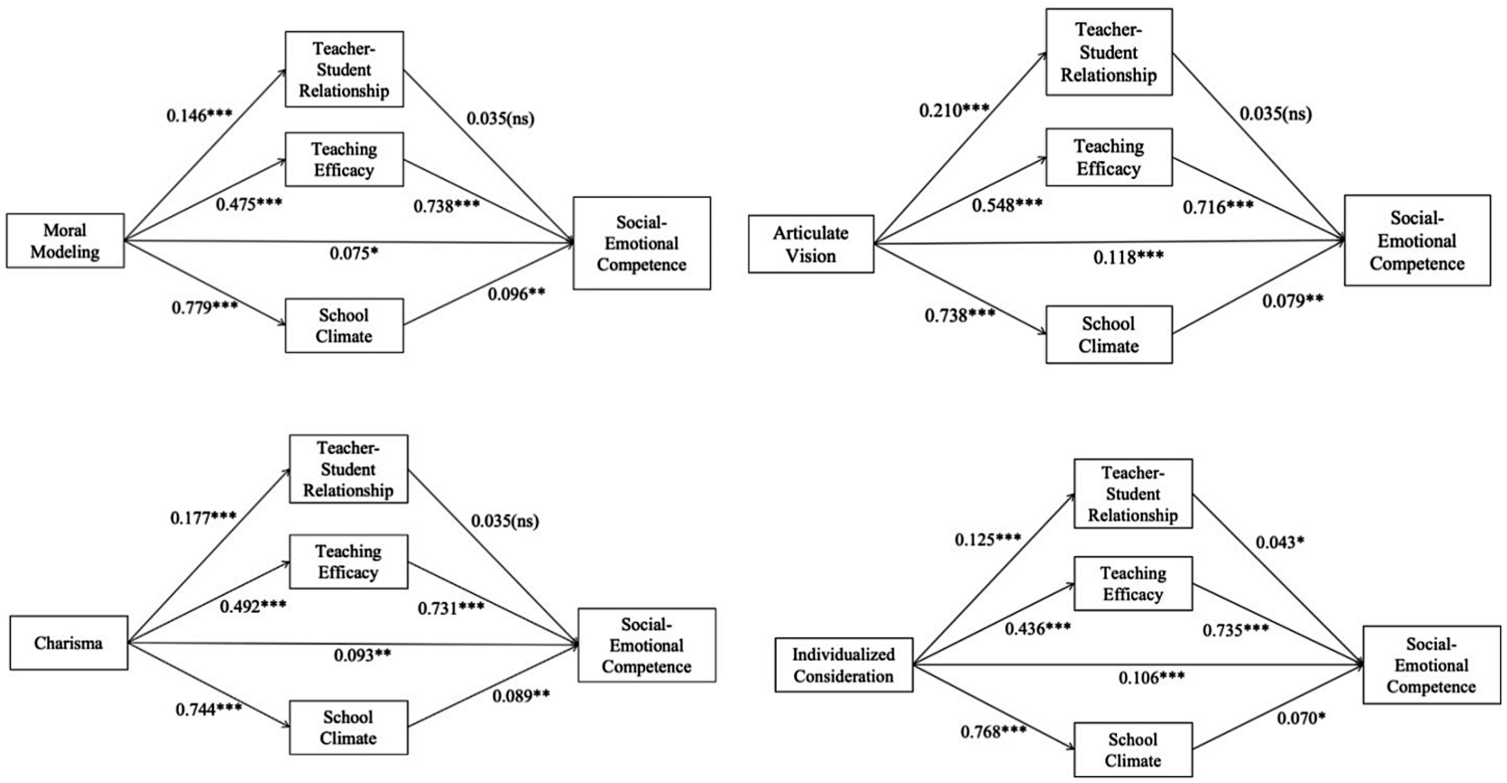
Multiple indirect associations between transformational leadership subdimensions and social–emotional competence. Figure presents standardized regression coefficients for the component paths in each dimension-specific mediation model. The statistical significance of the indirect associations was evaluated using bootstrap confidence intervals and is reported in Table 6. *p < 0.05; **p < 0.01; ***p < 0.001; ns = not significant.

To further examine whether these indirect-effect patterns varied across the four dimensions of Transformational Leadership, Moral Modeling, Articulate Vision, Charisma, and Individualized Consideration were entered separately as independent variables, and the same analytic procedures were repeated. Figure 3 presents the standardized regression coefficients for the component paths in each dimension-specific mediation model, and the detailed standardized coefficients are reported in Supplementary Tables S1–S4. Across all four models, Teaching Efficacy consistently showed a statistically significant positive regression coefficient with Social–Emotional Competence (standardized *β*s = 0.716–0.738). In contrast, the coefficients involving Teacher-Student Relationship and School Climate varied across the dimension-specific models.

For Moral Modeling, Articulate Vision, and Charisma, the estimated indirect effect via Teaching Efficacy was the largest (effects = 0.270, 0.317, and 0.274, respectively), and the estimated indirect effect via School Climate was also statistically significant (effects = 0.057, 0.047, and 0.051, respectively). By contrast, the indirect effect via Teacher-Student Relationship was not statistically significant in any of these three models. For Individualized Consideration, the estimated indirect effect via Teaching Efficacy remained statistically significant and was the largest (effect = 0.244, 95% CI = [0.185, 0.307]), whereas the indirect effects via Teacher-Student Relationship (95% CI = [−0.0001, 0.010]) and via School Climate (effect = 0.041, 95% CI = [−0.001, 0.081]) were not statistically significant. Detailed results are presented in Table 6. As an additional robustness check, we re-estimated the focal indirect association via Teaching Efficacy using a latent-variable mediation model based on the subdimensions of Transformational Leadership, Teaching Efficacy, and Social–Emotional Competence, while controlling for gender and age. This supplementary analysis was intended to examine whether the focal indirect association would remain evident when the three constructs were modeled as latent variables rather than observed composite scores. The indirect association remained statistically significant, and the overall pattern of the focal pathway was consistent with the main score-level analysis. Specifically, Transformational Leadership remained positively associated with Teaching Efficacy, Teaching Efficacy remained positively associated with Social–Emotional Competence, and the indirect association via Teaching Efficacy remained statistically significant in the latent-variable model. Detailed results are reported in Supplementary Table S7.

**Table 6:** Indirect effects of transformational leadership subdimensions on social–emotional competence.

Independent variable (subdimension)	Mediating pathway	Effect	Boot SE	95% CI lower limit	95% CI upper limit
1. Moral modeling	School climate	0.057	0.021	0.016	0.097
	Teacher-student relationship	0.004	0.003	−0.001	0.011
	Teaching efficacy	0.270	0.030	0.213	0.331
2. Articulate vision	School climate	0.047	0.019	0.011	0.086
	Teacher-student relationship	0.006	0.004	−0.002	0.014
	Teaching efficacy	0.317	0.031	0.258	0.379
3. Charisma	School climate	0.051	0.019	0.012	0.088
	Teacher-student relationship	0.005	0.003	−0.001	0.012
	Teaching efficacy	0.274	0.030	0.218	0.334
4. Individualized consideration	School climate	0.041	0.021	−0.001	0.081
	Teacher-student relationship	0.004	0.003	−0.0001	0.010
	Teaching efficacy	0.244	0.031	0.185	0.307

Bootstrap resampling = 5,000. Indirect effects are reported with bootstrapped standard errors and 95% confidence intervals.

## 5 Discussion

This study examined the association between Transformational Leadership and teachers’ Social–Emotional Competence and provided preliminary evidence regarding possible indirect association patterns involving Teacher-Student Relationship, Teaching Efficacy, and School Climate. The statistical results were consistent with H1, H2, and H3, provided only partial support for H5, whereas H4 was not supported in the overall model.

### 5.1 The association between transformational leadership and teachers’ social–emotional competence

The present study found that Transformational Leadership was significantly and positively associated with primary and secondary school teachers’ Social–Emotional Competence, which is consistent with Hypothesis 1 (H1). This finding indicates that higher reported levels of Transformational Leadership among school leaders were associated with higher reported levels of Social–Emotional Competence among teachers, which is broadly consistent with prior evidence (Tian et al., 2021a). Transformational Leadership exhibited by school leaders may be associated with both individual and collective functioning within the school environment, particularly in supportive relational and organizational contexts. In this respect, it may be related to school environments characterized by communication, support for teachers’ motivation and development, and more supportive social networks, all of which may in turn be associated with teachers’ Social–Emotional Competence (Xiong et al., 2023).

More specifically, supportive leadership behaviors may be associated with teachers’ sense of psychological safety and with fewer affective barriers related to traditional hierarchical management. When teachers report stronger perceptions of trust and support from leaders, they may also report greater use of emotion regulation strategies in their instructional practices. This pattern may be interpreted as being consistent with the possibility that teachers who reported more supportive leadership contexts also tended to report more active management of their emotions during teaching. To some extent, the present findings are broadly consistent with prior literature suggesting that Transformational Leadership has often been regarded as a highly valued leadership style (Yukl et al., 2002). Importantly, within educational settings shaped by collectivistic cultural norms in China, Transformational Leadership may be relevant not only to administrative functioning but also to teachers’ emotional and psychological experiences related to school life.

### 5.2 Indirect associations involving teacher-student relationship, teaching efficacy, and school climate

The three proposed indirect pathways were not equally supported in the present data. In the overall model, the estimated indirect effect via Teaching Efficacy was clearly the strongest and most consistent, the indirect effect via Teacher-Student Relationship was statistically significant but small, and the indirect effect via School Climate was not statistically significant.

First, the estimated indirect association via Teaching Efficacy was the largest in the overall model, which was consistent with Hypothesis 3 (H3). This finding is consistent with treating Teaching Efficacy as a proximal individual-level correlate of teachers’ Social–Emotional Competence. As a cognitive and motivational self-appraisal, Teaching Efficacy reflects teachers’ confidence in managing instructional demands and may be closely related to how teachers perceive and respond to the emotional and relational demands of their work (Zhang and Mao, 2022). Teachers who reported more supportive leadership contexts also tended to report stronger efficacy-related beliefs, and these beliefs were closely associated with their reported Social–Emotional Competence. Given the high correlation between Teaching Efficacy and Social–Emotional Competence, this result should be interpreted as the strongest estimated indirect association in the present model, rather than as evidence that Teaching Efficacy represents a fully independent construct-level contribution.

Second, the estimated indirect effect via Teacher-Student Relationship was statistically significant in the overall model, which was consistent with Hypothesis 2 (H2). This result may indicate that higher reported Transformational Leadership was associated with more positive reported Teacher-Student Relationship and that Teacher-Student Relationship was also associated with Social–Emotional Competence. However, the magnitude of this indirect effect was very small (indirect effect = 0.007), accounting for only 1.44% of the total effect, which suggests limited practical significance in the overall model despite its statistical significance. One possible reason is that Transformational Leadership and Teacher-Student Relationship are situated at different relational levels. Transformational Leadership primarily concerns organizational-level interactions between principals and teachers, whereas Teacher-Student Relationship unfolds within classroom-level interactions. From the perspective of ecological systems theory, associations observed at the school management level may not be strongly or consistently reflected in classroom-level relational patterns. Accordingly, this indirect effect should be interpreted cautiously and should not be regarded as a major indirect pattern in the overall model. Rather, Teacher-Student Relationship may represent only a supplementary and comparatively limited indirect association in the present data.

Third, the estimated indirect association via School Climate was not statistically significant in the overall model, and thus Hypothesis 4 (H4) was not supported at the overall level. Although Transformational Leadership showed a significant regression coefficient with School Climate, School Climate did not show a significant regression coefficient with Social–Emotional Competence after the three proposed mediators were considered simultaneously. Accordingly, School Climate should be interpreted as a less consistent statistical correlate in the overall model.

Taken together, the multiple mediation analyses indicated that the three estimated indirect associations differed in strength and stability within the present cross-sectional model. Teaching Efficacy showed the most prominent estimated indirect association, Teacher-Student Relationship showed a statistically significant but small indirect association, and School Climate was not supported in the overall model. These findings should not be interpreted as evidence of directional processes. Rather, they indicate that efficacy-related beliefs were more closely aligned with teachers’ reported Social–Emotional Competence than the other two proposed correlates under the specified statistical model.

### 5.3 Variation in indirect effects across the subdimensions of transformational leadership

Further supplementary dimension-specific analyses suggested some variation in the estimated indirect association patterns linking the four subdimensions of Transformational Leadership with teachers’ Social–Emotional Competence in models involving the three proposed mediators. These analyses were intended to clarify whether the indirect-effect pattern varied across leadership dimensions, but they should not be interpreted as overriding the overall model. Although all four subdimensions showed positive associations with teachers’ Social–Emotional Competence, the relative prominence of the indirect effects was not identical across models, which provided only partial support for Hypothesis 5 (H5).

First, the estimated indirect effect via Teaching Efficacy was the most consistently observed across the four subdimensions. In all four models, the path from Teaching Efficacy to Social–Emotional Competence remained statistically significant (standardized *β*s = 0.716–0.738), and the bootstrap indirect effect via Teaching Efficacy was statistically significant in every model. This pattern suggests that, within the present data, Teaching Efficacy showed the most stable estimated indirect association among the three proposed mediators across the four subdimensions of Transformational Leadership. At the same time, this pattern should be interpreted as a relatively stable proximal association rather than as evidence of a sharply distinct indirect pattern.

Second, the estimated indirect association involving School Climate showed a dimension-specific pattern. Although the School Climate pathway was not significant in the overall model, statistically significant indirect associations via School Climate were observed for Moral Modeling, Articulate Vision, and Charisma, but not for Individualized Consideration. One possible interpretation is that Moral Modeling, Articulate Vision, and Charisma were more closely aligned with teachers’ broader perceptions of the school environment in this sample, whereas Individualized Consideration showed a less consistent pattern with respect to climate-related perceptions. This interpretation remains tentative and should be viewed as supplementary.

Finally, the indirect effect via Teacher-Student Relationship showed a generally weak and non-robust pattern in the dimension-specific analyses. In the overall model, the indirect effect via Teacher-Student Relationship was statistically significant but accounted for only a very small proportion of the total effect, suggesting that it represented a comparatively limited indirect association. In the subdimension analyses, Teacher-Student Relationship did not show statistically significant indirect effects for Moral Modeling, Articulate Vision, Charisma, or Individualized Consideration, as all confidence intervals included zero. This pattern suggests that the indirect association via Teacher-Student Relationship was not consistently evident across the four subdimensions in the present data. A cautious interpretation is that this indirect pattern may depend on teacher-level or classroom-level conditions that were not fully captured in the current model. Overall, the present findings indicate that the indirect effect via Teaching Efficacy was the most consistent across subdimensions, whereas the indirect effect via School Climate appeared more conditional and dimension-specific, and the indirect effect via Teacher-Student Relationship appeared comparatively limited.

### 5.4 Limitations and future directions

This study examined the indirect association patterns linking Transformational Leadership with primary and secondary school teachers’ Social–Emotional Competence. Although the findings provide several useful insights, the study remains subject to limitations that should be addressed in future research. First, as with other cross-sectional studies, this research is subject to the usual limitations regarding causal inference. Because data were collected at a single time point, the model results support statistically significant associations among variables, yet they do not allow for strict conclusions about causal direction. Future research could adopt longitudinal designs or cross-lagged panel analyses by collecting data at multiple time points to further clarify how Transformational Leadership and teachers’ Social–Emotional Competence are associated over time. In addition, all core variables in this study were collected through teachers’ self-report at a single time point, which may have increased the risk of common method bias. Harman’s single-factor test was used only as a preliminary diagnostic check. Given that all core variables were collected through same-source self-report measures at a single time point, this procedure provides only limited evidence regarding common method bias and cannot meaningfully rule out method-related inflation of the observed associations. Moreover, because Teaching Efficacy and Social–Emotional Competence were strongly related and were both measured through same-source self-report data, the estimated indirect association via Teaching Efficacy may partly reflect shared self-evaluative variance. Future research should use longitudinal, multi-source, and more rigorously validated measurement designs to further clarify the distinctiveness and temporal ordering of these constructs.

Second, regarding sample representativeness, due to resource constraints, this study employed convenience sampling. Participants were primarily drawn from primary and secondary schools in the Beijing-Tianjin-Hebei region and surrounding areas, and the proportion of female teachers was relatively high. These characteristics may, to some extent, limit the external validity and generalizability of the findings. Future studies should expand the sampling frame and adopt more rigorous sampling strategies. The dimension-specific patterns should also be interpreted cautiously and examined in future samples.

Finally, this study primarily focused on indirect associations and did not examine potential moderators in depth. Variables such as teachers’ years of teaching experience, emotional intelligence, and organizational structural characteristics of schools may represent important contextual conditions associated with the patterns observed here. Future research could further investigate, at both individual and organizational levels, whether and how these factors moderate the associations involving leadership behaviors.

## Data Availability

The raw data supporting the conclusions of this article will be made available by the authors, without undue reservation.
