# Supplementary material for: How transformational leadership is associated with primary and secondary school teachers’ social–emotional competence: a multiple mediation analysis
Source: Front Psychol. 2026 Jun 3;17:1777257. doi: 10.3389/fpsyg.2026.1777257 (PMC13272482; doi:10.3389/fpsyg.2026.1777257)
Supplement: Supplementary file 1 [file Table_1.DOC]

**How Transformational Leadership is Associated with Primary and Secondary School Teachers’ Social-Emotional Competence: A Multiple Mediation Analysis**

**Supplementary Materials**

Table S1. Standardized Regression Coefficients for the Moral Modeling (TLQ1) Model

| Outcome Variable | Predictor | β | SE | t | p |
| --- | --- | --- | --- | --- | --- |
| Teacher-Student Relationship | TLQ1 | 0.146 | 0.026 | 4.008 | < .001 |
| Teaching Efficacy | TLQ1 | 0.475 | 0.025 | 18.991 | < .001 |
| School Climate | TLQ1 | 0.779 | 0.012 | 33.765 | < .001 |
| Social-Emotional Competence | TLQ1 | 0.075 | 0.024 | 2.376 | .018 |
| Social-Emotional Competence | Teacher-Student Relationship | 0.035 | 0.023 | 1.696 | .090 |
| Social-Emotional Competence | Teaching Efficacy | 0.738 | 0.024 | 32.313 | < .001 |
| Social-Emotional Competence | School Climate | 0.096 | 0.049 | 2.998 | .003 |

Table S2. Standardized Regression Coefficients for the Articulate Vision (TLQ2) Model

| Outcome Variable | Predictor | β | SE | t | p |
| --- | --- | --- | --- | --- | --- |
| Teacher-Student Relationship | TLQ2 | 0.210 | 0.027 | 5.736 | < .001 |
| Teaching Efficacy | TLQ2 | 0.548 | 0.023 | 18.675 | < .001 |
| School Climate | TLQ2 | 0.738 | 0.013 | 28.977 | < .001 |
| Social-Emotional Competence | TLQ2 | 0.118 | 0.024 | 3.995 | < .001 |
| Social-Emotional Competence | Teacher-Student Relationship | 0.035 | 0.022 | 1.742 | .082 |
| Social-Emotional Competence | Teaching Efficacy | 0.716 | 0.024 | 30.352 | < .001 |
| Social-Emotional Competence | School Climate | 0.079 | 0.043 | 2.820 | .005 |

Table S3. Standardized Regression Coefficients for the Charisma (TLQ3) Model

| Outcome Variable | Predictor | β | SE | t | p |
| --- | --- | --- | --- | --- | --- |
| Teacher-Student Relationship | TLQ3 | 0.177 | 0.025 | 4.818 | < .001 |
| Teaching Efficacy | TLQ3 | 0.492 | 0.023 | 16.142 | < .001 |
| School Climate | TLQ3 | 0.744 | 0.012 | 29.674 | < .001 |
| Social-Emotional Competence | TLQ3 | 0.093 | 0.022 | 3.203 | .001 |
| Social-Emotional Competence | Teacher-Student Relationship | 0.035 | 0.022 | 1.705 | .089 |
| Social-Emotional Competence | Teaching Efficacy | 0.731 | 0.024 | 31.936 | < .001 |
| Social-Emotional Competence | School Climate | 0.089 | 0.044 | 3.092 | .002 |

Table S4. Standardized Regression Coefficients for the Individualized Consideration (TLQ4) Model

| Outcome Variable | Predictor | β | SE | t | p |
| --- | --- | --- | --- | --- | --- |
| Teacher-Student Relationship | TLQ4 | 0.125 | 0.025 | 3.420 | .001 |
| Teaching Efficacy | TLQ4 | 0.436 | 0.023 | 14.059 | < .001 |
| School Climate | TLQ4 | 0.768 | 0.012 | 33.053 | < .001 |
| Social-Emotional Competence | TLQ4 | 0.106 | 0.023 | 3.472 | .001 |
| Social-Emotional Competence | Teacher-Student Relationship | 0.043 | 0.023 | 2.072 | .039 |
| Social-Emotional Competence | Teaching Efficacy | 0.735 | 0.023 | 32.941 | < .001 |
| Social-Emotional Competence | School Climate | 0.070 | 0.048 | 2.202 | .028 |

Note. Standardized regression coefficients are reported. The same demographic covariates as in the main mediation model were included but are not displayed for brevity.

Table S5. Fit indices for the CFA models of Teaching Efficacy and Social-Emotional Competence

| Model | χ² | df | CFI | TLI | RMSEA | SRMR |
| --- | --- | --- | --- | --- | --- | --- |
| One-factor model | 6121.645 | 1034 | .892 | .887 | .080 | .056 |
| Two-factor model | 5489.700 | 1033 | .905 | .901 | .075 | .053 |
| Revised two-factor model | 4740.788 | 1027 | .921 | .917 | .069 | .050 |

Note. One-factor model = all Teaching Efficacy and Social-Emotional Competence items loaded on a single latent factor. Two-factor model = Teaching Efficacy and Social-Emotional Competence specified as two correlated latent factors. Revised two-factor model = two-factor model with a small number of theory-guided residual correlations among similarly worded items within the same construct. All models were estimated in Mplus using the WLSMV estimator.

Table S6. Additional model-comparison evidence for the empirical distinctiveness of Teaching Efficacy and Social-Emotional Competence

| Model / Statistic | Value |
| --- | --- |
| Restrictive model imposing perfect association: χ² | 6121.645 |
| Restrictive model imposing perfect association: df | 1034 |
| Restrictive model imposing perfect association: CFI | .892 |
| Restrictive model imposing perfect association: TLI | .887 |
| Restrictive model imposing perfect association: RMSEA | .080 |
| Restrictive model imposing perfect association: SRMR | .056 |
| DIFFTEST: Δχ² | 312.498 |
| DIFFTEST: Δdf | 1 |
| DIFFTEST: p | < .001 |
| Freely estimated two-factor model: latent correlation (TE with SEC) | .914 |

Note. The freely estimated two-factor model allowed the latent association between Teaching Efficacy and Social-Emotional Competence to be estimated freely. The restrictive model imposed perfect association between the two latent variables. The DIFFTEST result compares the restrictive model with the freely estimated two-factor model. In the present specification, imposing a perfect association between the two latent variables yielded the same global fit indices as the one-factor model.

Table S7. Focused latent-variable mediation robustness check for the indirect association via Teaching Efficacy

| Path / Statistic | Estimate | 95% CI |
| --- | --- | --- |
| CFI | .948 | — |
| TLI | .936 | — |
| RMSEA | .088 | — |
| SRMR | .059 | — |
| TE ON TL (STDYX β) | .595 | — |
| SEC ON TE (STDYX β) | .874 | — |
| SEC ON TL (STDYX β) | .127 | — |
| TL → TE → SEC (unstandardized indirect effect) | .366 | [.288, .458] |
| TL → TE → SEC (standardized indirect effect) | .520 | [.445, .599] |

Note. This model was estimated as a focused latent-variable robustness check based on the subdimensions of Transformational Leadership, Teaching Efficacy, and Social-Emotional Competence, while controlling for gender and age. The model was not intended to replace the main multiple-mediation model, but to examine whether the focal indirect association via Teaching Efficacy remained statistically significant in a latent-variable framework. Reported coefficients are standardized (STDYX) unless otherwise noted. The indirect effect remained statistically significant, and the overall pattern was consistent with the main score-level analysis.
